# European basic laparoscopic urological skills: a feasibility study in a setting for robot-assisted surgery

**DOI:** 10.3389/fsurg.2025.1566840

**Published:** 2025-04-14

**Authors:** Nikolaos Liakos, Martin Janssen, Rudolf Moritz, Özlem Kayaci-Güner, Johannes Bründl, Arif Özkan, Burkhard Ubrig, Stefan Siemer, Christian Gratzke, Christian Wagner

**Affiliations:** ^1^ Department of Urology, Faculty of Medicine, Medical Centre of the University of Freiburg, Freiburg, Germany; ^2^ Department of Urology, Faculty of Medicine, Medical Centre of the University of Münster, Münster, Germany; ^3^ Department of Urology, Marien Hospital Herne, University Hospital of the Rhein-Ruhr-University, Herne, Germany; ^4^ European Robotic Institute, Gronau, Germany; ^5^ Department of Urology, St. Josef Krankenhaus Regensburg, University Hospital of the University of Regensburg, Regenburg, Germany; ^6^ Department of Urology, Augusta-Kranken-Anstalt Bochum, University Hospital of the Rhein-Ruhr-University, Bochum, Germany; ^7^ Department of Urology, Faculty of Medicine, Medical Centre of the University of Saarland, Homburg/Saar, Germany; ^8^ Department of Urology, Urologic Oncology and Robot-Assisted Surgery, St. Antonius Hospital Gronau, Gronau, Germany

**Keywords:** E-BLUS, robotics, simulation, surgical education, robotic training

## Abstract

**Introduction:**

Since the introduction of laparoscopy, a variety of training sets and tasks have been introduced for surgical education of minimally-invasive surgery. The implementation of the European Basic Laparoscopic Urological Skills into the training and education program of future laparoscopic surgeons created a new era and provided a standardized approach for urological surgical training. However, these tasks have not yet been evaluated in a setting of robot-assisted surgery. The aim of this study is to evaluate the feasibility of the implementation of the four E-BLUS tasks into training modules of robot-assisted surgery.

**Methods:**

A cohort of 31 robotic surgeons (group A: experienced, group B: novices) performed these tasks in two different institutions by using the latest generation of robotic surgical platforms. Time performance and failure rate were assessed and statistically analyzed.

**Results:**

The groups demonstrated a statistically significant difference regarding time performance in half of the tasks involving fine surgical skills (cutting and knotting, *p* = 0.01 and *p* = 0.02, respectively) but no significant difference in tasks involving manual ambidexterity (*p* = 0.14 and 0.12, respectively). A low failure rate during the attempts of the group of novice robotic surgeons could be observed.

**Discussion:**

The use of the E-BLUS tasks in a training setting of robot-assisted surgery is feasible and can lead to the development of surgical skills needed during robot-assisted surgical procedures. It is a relatively low-cost dry lab option for the introduction of novice robotic surgeons.

## 1 Introduction

The introduction of laparoscopic transperitoneal nephrectomy in the early 1990 s manifested a breakthrough in the urological discipline (1). Since the introduction of laparoscopy as an option for various surgical treatments of urological diseases, there was the need for further simulation training for the purpose of dissemination of this novel surgical option. As the principle of Halsted could not be the way of achieving expertise (“see one, do one, teach one”), many training programs for the development of basic as well as intermediate skills have been proposed and evaluated (2). The implementation of these programs offered a potential for standardized and proficient way of teaching new laparoscopic surgeons, mainly offering the essential advantage of being able to repeat the designated exercises in a simulation setting, thus optimizing the laparoscopic skills without exposing the patient to risks associated with absence of laparoscopic experience (3, 4).

Beginning with the Fundamentals of Laparoscopic Surgery (FLS) in the United States of America, a program based on the inanimated training system of the McGill University for teaching and evaluating laparoscopic skills, a training program for Laparoscopy was introduced to the surgical societies and underwent thorough evaluation and validation (5, 6). Due to the differences to the American Training and Residency program, any developed variation for the European setting should undergo validation. Thus, the Program for Laparoscopic Urological Skills (PLUS) was introduced after respective validation (7). Working at the fundamentals of this validated program, Veneziano et al. (8) created the European Basic Laparoscopic and Urological Skills (E-BLUS) program and introduced this program through the various meeting and congresses of the European Association of Urology (EAU) (8, 9).

The actual version of the E-BLUS practical examination consists of four different laparoscopic tasks in a pelvitrainer with a laparoscopic camera fixed on a predefined position (10). The examination includes the following four tasks: peg transfer, cutting the circle, needle guidance and single knot tying. Through these tasks, competences such as bimanual dexterity, hand-eye-coordination, spatial awareness and technical skills such as cutting and suturing can be assessed (11). During the first task, six small pegs have to be transferred from the non-dominant to the dominant side (depending on handedness) and vice versa, without letting any pegs to fall from the instruments after grasping them, and to placie them at the predefined rods. During the second task (cutting the circle), a circle delineated with two black circular lines printed on a surgical gauze must be cut out, without violating the integrity of these lines. At task 3 (needle guidance), a needle must be passed through ten mini circles using both instruments (hands), in a predefined track. At single knot tying, the examinee must form a simple knot by placing a suture at a small piece of incised Penrose drainage, by placing the needle through two predefined dots already marked on the side of the incision at the drainage (8).

Up to the present time, there has not been any study considering the feasibility of these training tasks in a setting of robot-assisted surgery (12). The aim of this study is to perform the first known feasibility assessment of the E-BLUS task in a training setting of robot-assisted surgery.

## 2 Material and methods

Between October 2022 and July 2023, an overall cohort of 31 urological surgeons were assessed, divided into two different groups: group A with five (*n* = 5) experienced robotic surgeons each one with an experience of more than 500 cases and group B with 26 novice robotic surgeons (*n* = 26) with no or minimal robotic experience (each one <10 cases as first surgeon in his/her career). All novice surgeons mentioned no previous laparoscopic experience as a first surgeon. The assessment took place in two different centers (Strasbourg, France and Münster, Germany) and two different tutors for robotic surgery supervised the completion of the tasks for both groups. For the purpose of the study, robotic surgical platforms of the fourth generation of the Da Vinci System were used (Da Vinci X and Xi systems; Intuitive Surgical, Sunnyvale, CA, USA).

We calculated the average time for completion of each task with the respective interquartile range as well as the frequency of failing to reach the pass benchmark of each task, as it has been predefined by the EAU. The results of the evaluation were systematically documented and statistically analyzed using the SPSS software (SPSS Statistics, Version 29, IBM, Armonk, NY, USA). Fisher's exact test was used of the categorical variables and the unpaired *t*-test was used for continuous variables. Statistically significant difference between the values was determined if *p* < 0.05.

To guarantee non-interference of the various training boxes to the quality of the data being documented during the assessment, we used the E-BLUS kit provided by the European School of Urology and the training dome provided for robot-assisted training from Intuitive Surgical™ (Sunnyvale, CA, USA). Due to the fact of inadequate experience in robot-assisted surgery in group B (novices) we did not evaluate the assessment with a pass/fail mark.

## 3 Results

**Figure 1:**
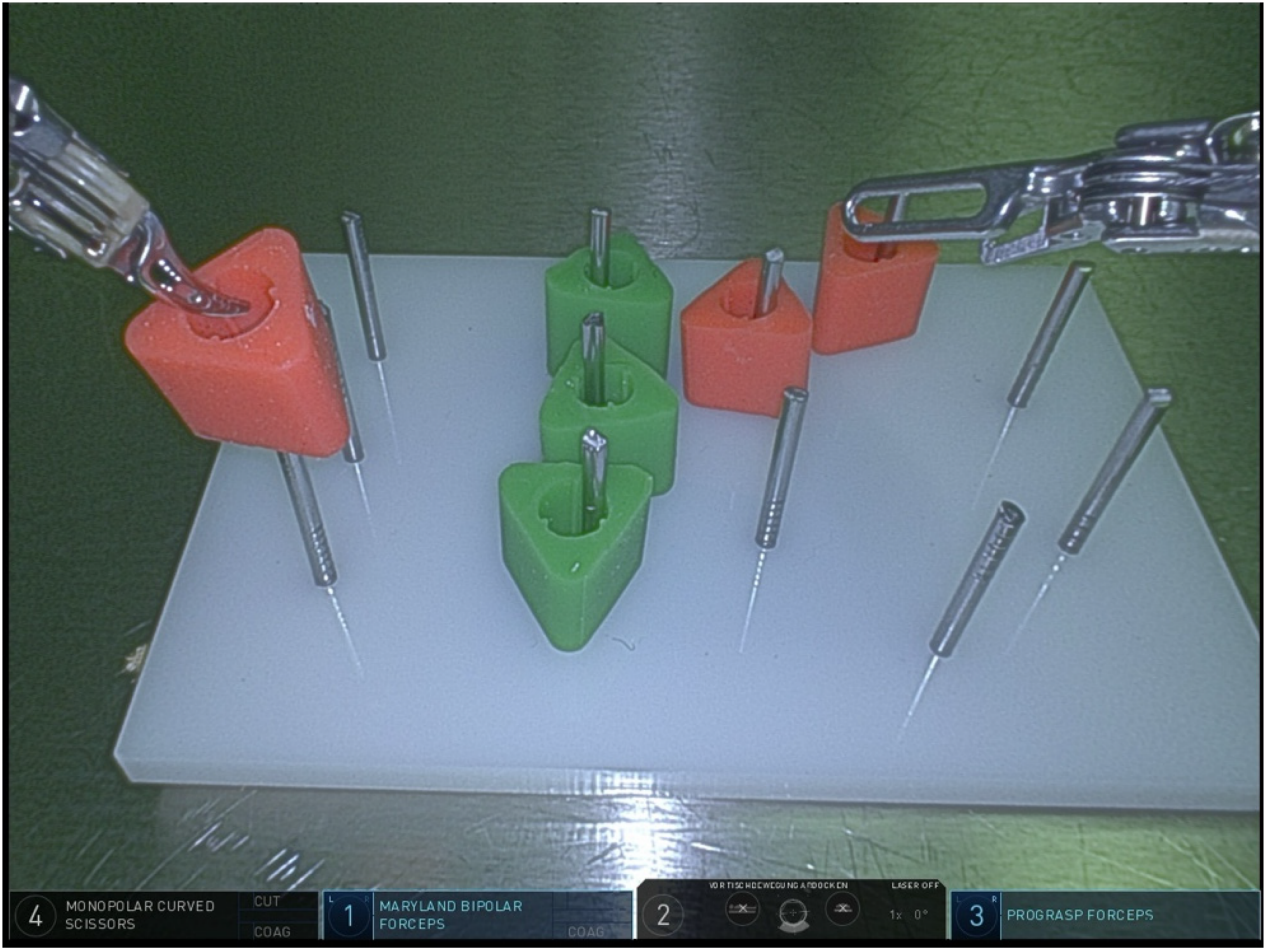
Task 1 (peg transfer).

Regarding task 1 (peg transfer, Figure 1), group A had a mean completion time of 84.5 s (range 37–137 s, SD 57.9 s) in contrast to group B with an average completion time of 151.7 s (range 76–560 s, SD 29.7 s, average time addition of 79.5%). The difference between the groups was not statistically significant (*p* = 0.14, results demonstrated in Table 1 and Graphic 1). In group B, there was the failure rate of 15.4% (four participants) in this task during the first attempt by losing (involuntarily dropping) the peg due to handling failure, whereas in the group of experts, all study participants accomplished the task.

**Table 1:** Task 1 (peg transfer)—completion time & failure rate.

	Group A	Group B
Average completion time (sec)	85.4 (SD 57.9)	151.69 (SD 29.7)
Median completion time (sec)	69	127
Q1	65	100
Q3	119	151.5
IQR	54	41.5
*p*	0.14	
Failure rate	0%	16.13%

IQR, interquantile range; Q1, quartile 1; Q3, quartile 3.

**Figure 2:**
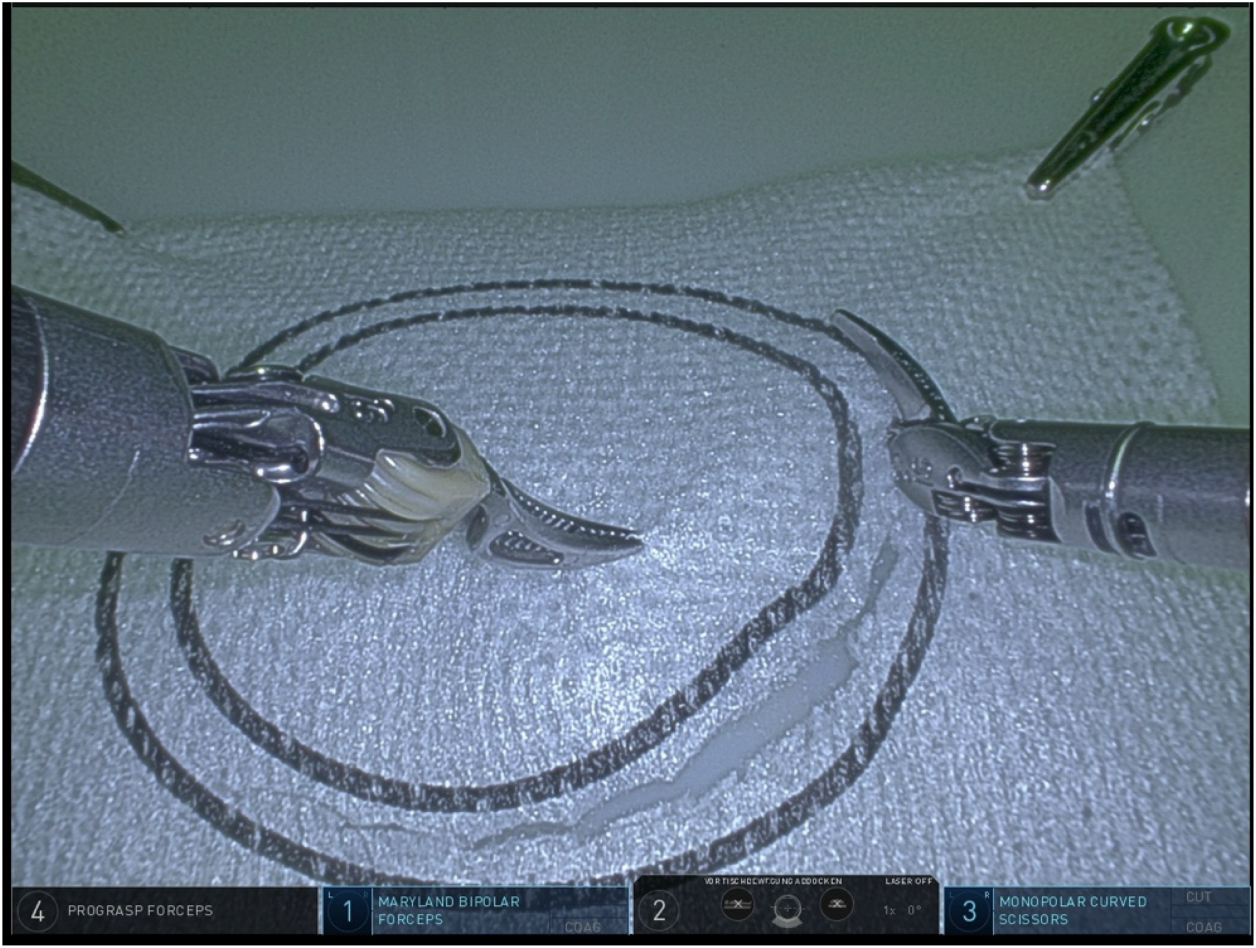
Task 2 (cutting the circle).

Group A demonstrated an mean completion time of 60.8 s (range 48–88 s, SD 27.8 s) to accomplish task 2 (cutting the circle, Figure 2), group B had an average completion time of 164.46 s (range 87–524 s, SD 10.6 s, time addition of 170.5%), presenting a statistically significant difference (*p* = 0.01, results demonstrated in Table 2 and Graphic 2). In group B, the failure rate of the task by disturbing the continuity of the circles was 30.8% during their attempts. In group A, there were no failed attempts.

**Table 2:** Task 2 (cutting the circle)—completion time & failure rate.

	Group A	Group B
Average completion time (sec)	60.8 (SD 27.8 s)	164.46 (SD 10.6 s)
Median completion time (sec)	49	156.5
Q1	112	49
Q3	179	70
IQR	67	21
*p*	0.01	
Failure rate (line alteration)	0%	30.8%
*p*	0.29	

**Figure 3:**
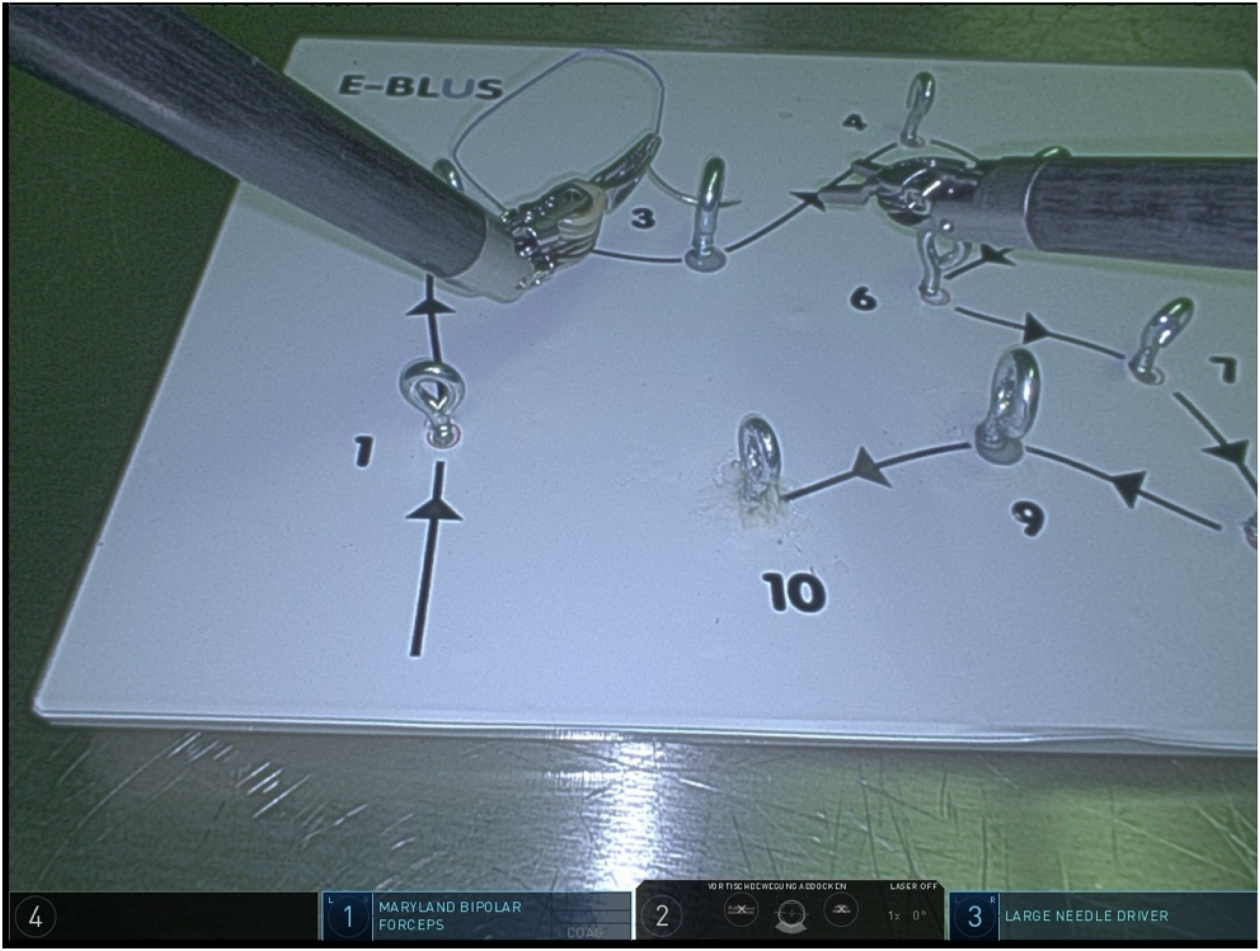
Task 3 (needle guidance).

Regarding task 3 (needle guidance, Figure 3) group A demonstrated a mean completion time of 73.2 s (range 45–101 s, SD 24.8 s), whereas the group of novices demonstrated an average completion time of 165.06 s (range 84–755 s, SD 6.4 s, average time addition of 124.6%). There was no statistically significant difference between the two groups (*p* = 0.12, results demonstrated in Table 3 and Graphic 3).

**Table 3:** Task 3 (needle guidance)—completion time & failure rate.

	Group A	Group B
Average completion time (sec)	73.2 (SD 24.8 s)	165.06 (SD 6.4 s)
Median completion time (sec)	62	138.5
Q1	61	116.25
Q3	97	158
IQR	36	41.75
*p*	0.19	

**Figure 4:**
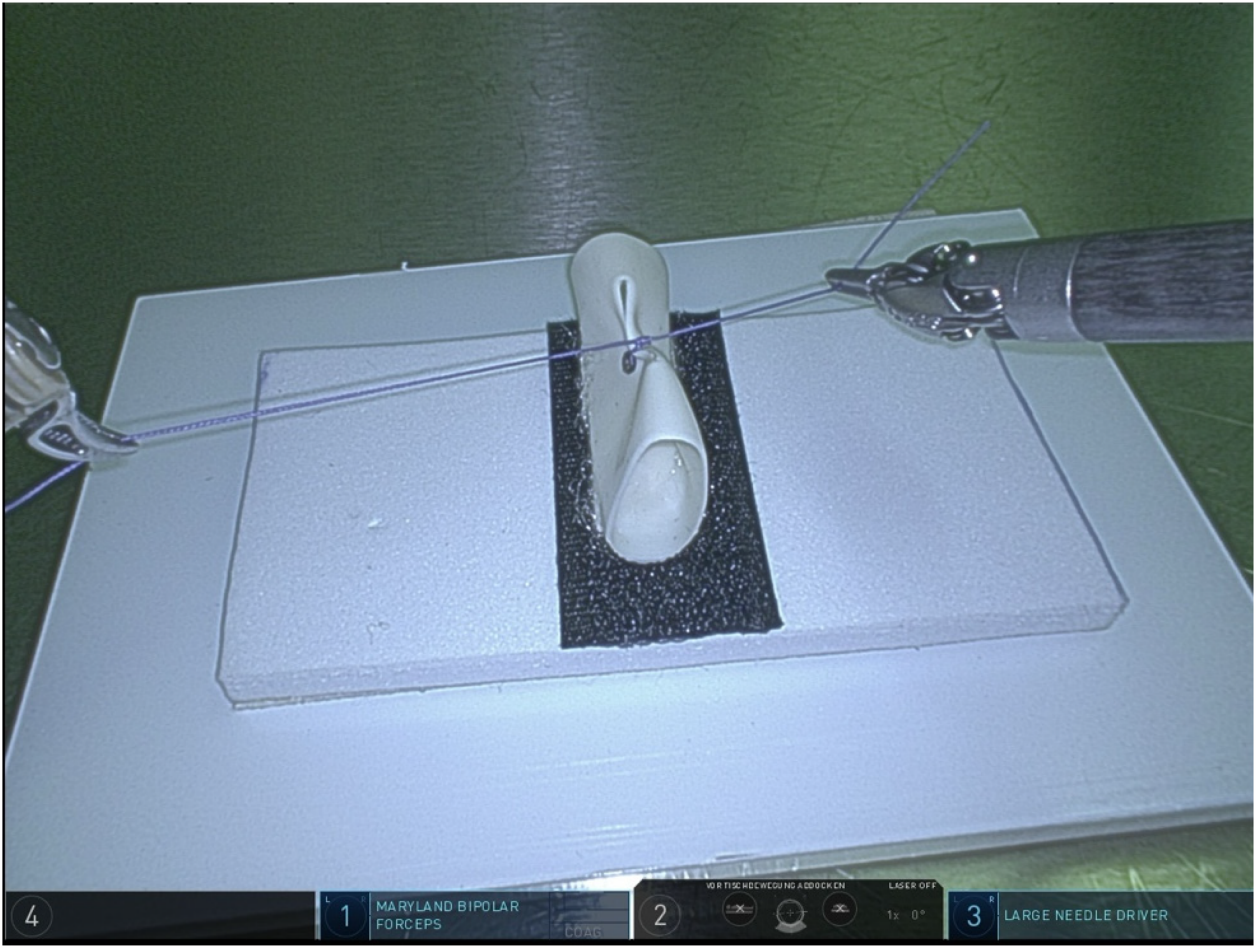
Task 4 (simple knot tying).

Regarding task 4 (single knot tying, Figure 4), groups A and B had a mean time of completion of 92 s and 182.46 s, respectively (SD 1.4 s and 19.1 s respectively, average time addition of 98.3%). The time difference between the two groups was statistically significant (*p* = 0.02, results demonstrated in Table 4 and Graphic 4). Group A had a failure rate of 20% (1/5) after examining the details of the knot, particularly the placement of the stitch at the two predefined reference points. Group B had a failure rate of 3.9% regarding this aspect, a failure rate of 11.5% regarding the approximation of the two margins of the Penrose drainage and a 15.4% failure rate regarding the undesired, yet possible slipping of the knot, whereas the whole of group A examinees successfully passed the evaluation of the latter two aspects and met all desired quality criteria.

**Table 4:** Task 4 (simple knot tying)—completion time & failure rate.

	Group A	Group B
Average completion time (sec)	92 (SD 1.4 s)	182.42 (SD 19.1 s)
Median completion time (sec)	82	162.5
Q1	80	118
Q3	94	216.5
IQR	14	98.5
*p*	0.02	
Failure rate (suture placement)	20%	3.9%
*p*	0.31	
Failure rate (margin approximation)	0%	11.5%
*p*	1	
Failure rate (knot slipping)	0%	15.4%
*p*	1	

## 4 Discussion

Since the introduction of robot-assisted surgery, many training models as well as training strategies have been evaluated, implemented, revised or (early or later) rejected (13, 14). A plethora of dry and wet lab training tasks have been proposed, as a mean of early introduction into the console surgical exposure, prior to the on-the-patient surgical exposure (13). Many of the models for the training in robot-assisted surgery derive from the laparoscopic settings, which were developed and implemented years ago (15, 16).

Surprisingly, the E-BLUS tasks did not follow this rule. Although they have been presented in various (preliminary) forms (e.g., PLUS variant) and validated for the use in laparoscopic dry lab training, they have not been tested and evaluated in a robot-assisted surgical setting. In this study, we assessed the possibility of implementing the E-BLUS tasks in this particular setting. The results provided by our assessment demonstrate the feasibility of integrating a pure laparoscopic training module into the framework of training novice robotic surgeons.

The alternative to the laparoscopic training module integrated into the setting of robot-assisted surgery is the robotic console simulator of the system developer, a platform offering various tasks to train the capabilities of the future surgeons. Besides the high costs, the robotic console simulator remains a virtual reality simulator and does not fulfill all the needs of training prior to the console exposure. Therefore, we insist on the trainee's exposure to the laparoscopic pelvitrainer, whereby the future console surgeon can develop the manual dexterity, the spatial awareness and hand-eye coordination in a setting with tangible training objects.

The collected data regarding the results are concurring with the expectation of efficient, quicker and successful accomplishment of the tasks from the group of experienced surgeons (group A). As we only studied the feasibility of the tasks, we can only assess the performance in terms of time and failure to achieve the desired result. The assessment of bimanual dexterity, hand-eye coordination or three-dimensional spatial awareness were not objectives of the study. We observed a shorter mean time of completion of each task in the group of experienced surgeons in all four tasks of the assessment. There was a statistically significant difference between the two groups in tasks 2 (cutting the circle) and 4 (simple knot tying), *p* = 0.01 and *p* = 0.02 respectively. We consider these results as plausible, as for these two tasks, the necessary technical skills to accomplish these tasks are acquired after prolonged exposure at training and surgery performing settings (4).

We observed a rather low but indeed existing failure rate at all tasks performed by the novice robotic surgeons. This can be easily explained, as all these surgeons did not have the opportunity of a long exposure at training models or training facilities in their carriers. However, the failure rate of 20% (one of five) in the experienced group during task 4 (single knot tying) does not comply with the awaited level of experience of these surgeons. We presume that the surgeon proceeded to the completion of the tasks without bearing in mind that an error could be possible despite his experience (overconfidence). Moreover, the cohort of surgeons in group A was undersized, thus any failure during the task contributing a significant change in our study analysis.

The main limitation of our study is the need of a robotic system to perform the tasks, as such a robotic system is usually limited in availability, thus it is usually provided within the setting of a dedicated training center, alternatively the (clinical) robotic platform of a surgical center can be used after-hours. Moreover, there are existing costs to implement this dedicated training into the standardized surgical training program of new console surgeons (E-BLUS kit, approximately €50, as well as new gauzes and sutures for the simulation); additional costs cover the training instruments that have to be present for the tasks (scissors, needle driver, one variety of grasper). A further statistical limitation of our study is the small number of participants as well as the unequal number of surgeons allocated in both groups.

We all bear in mind that these tasks cannot replace all previous models of dry-lab training in a setting of robot-assisted training. Nevertheless, it is essential to provide various options for new robotic surgeons, especially at the beginning of their training sessions. It is common sense among the members of the surgical community that surgical skills cannot only be acquired by performing surgery but also beforehand, in an appropriate training setting (3). This set of surgical tasks can act as a primary set of training exercises for new console surgeons, at the very beginning of their education. Skills as suturing, cutting and suturing knot tying can be acquired through previously validated and easy to reproduce tasks (3, 17). Following and accomplishing simple tasks in a training setting can establish appropriate knowledge in less time, thus potentially minimizing the need of e.g., cadaveric models later in the training process.

Moreover, these tasks can also be used as a filtering mechanism in the process of trainee selection. Through these tasks we can assess the surgeons interested in training in robotics and select the people with potential in evolving their surgical skills. This proposal however should not be used as an axiom, that people underperforming in these tasks were not suitable for performing decent, above-average or outstanding robotic surgery in their later carrier.

## 5 Conclusion

The use of simple tasks, already implemented in the training structures of laparoscopic surgery, can be adopted in the training setting of robot-assisted surgery. Optimizing the surgical skills and the training time of novice console-surgeons can have a later positive effect on the patient outcomes. We demonstrated the feasibility of adoption of an already established and validated training set of laparoscopic tasks into the setting of robot-assisted surgery. By using these tasks, future console surgeons can acquire basic surgical skills in their early training phase without the need of on-patient training or the use of cadaveric models.

## Data Availability

The original contributions presented in the study are included in the article/Supplementary Material, further inquiries can be directed to the corresponding author.
