# Supplementary material for: European basic laparoscopic urological skills: a feasibility study in a setting for robot-assisted surgery
Source: Front Surg. 2025 Apr 14;12:1566840. doi: 10.3389/fsurg.2025.1566840 (PMC12034741; doi:10.3389/fsurg.2025.1566840)
Supplement: Supplementary file 1 [file Datasheet1.docx]

# Appendix

## Graphics

## Group A (experienced): blue – group B (novices): red

##
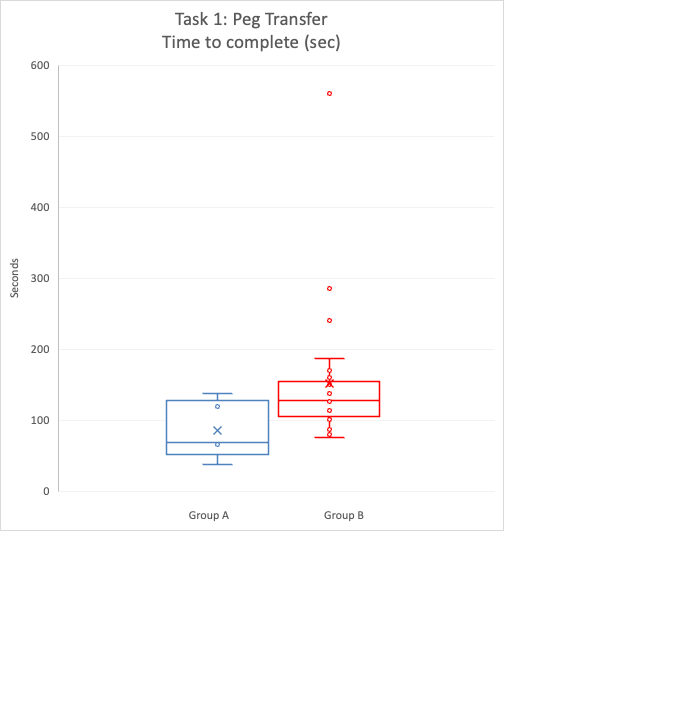


## Graphic 1: Task 1 (peg transfer) - time to complete


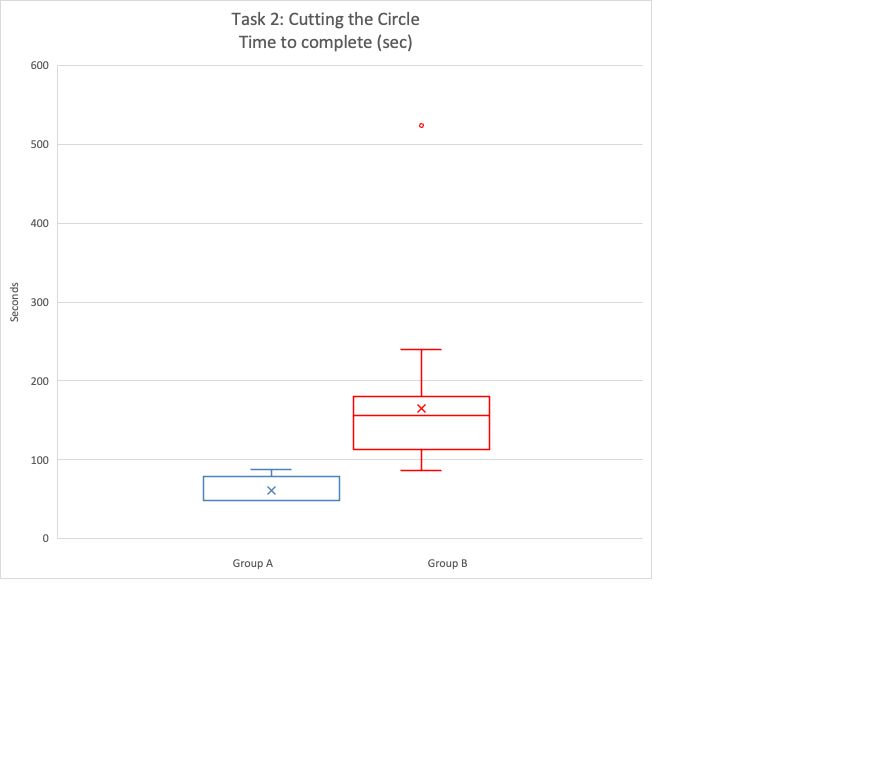


Graphic 2: Task 2 (cutting the circle) - time to complete


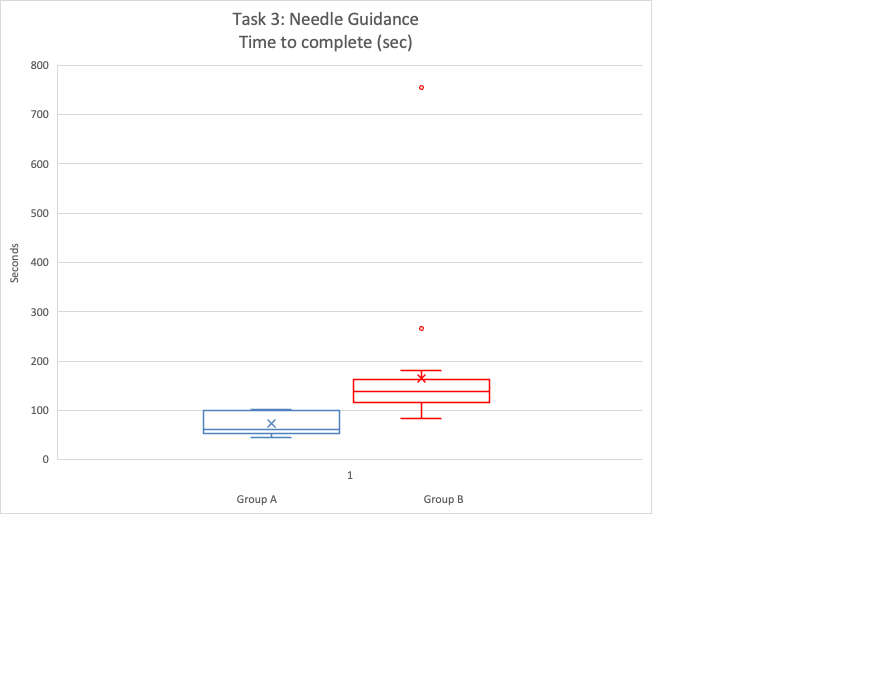


Graphic 3: Task 3 (needle guidance) - time to complete


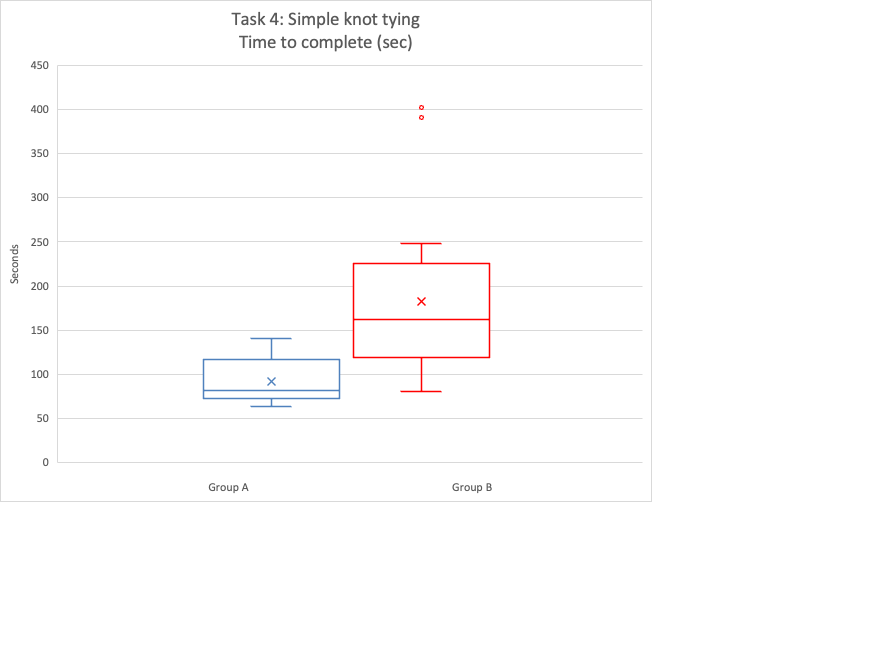


Graphic 4: Task 4 (simple knot tying) - time to complete

## Tables

IQR: interquantile range

Q1: quartile 1

Q3: quartile 3

Table 1: Task 1 (peg transfer) - completion time & failure rate.

|  | Group A | Group B |
| --- | --- | --- |
| Average completion time (sec) | 85.4 (SD 57.9) | 151.69 (SD 29.7) |
| Median completion time (sec) | 69 | 127 |
| Q1 | 65 | 100 |
| Q3 | 119 | 151.5 |
| IQR | 54 | 41.5 |
| p | 0.14 | |
| Failure rate | 0% | 16.13% |

Table 2: Task 2 (cutting the circle) - completion time & failure rate

|  | Group A | Group B |
| --- | --- | --- |
| Average completion time (sec) | 60.8 (SD 27.8 sec) | 164.46 (SD 10.6 sec) |
| Median completion time (sec) | 49 | 156.5 |
| Q1 | 112 | 49 |
| Q3 | 179 | 70 |
| IQR | 67 | 21 |
| p | 0.01 | |
| Failure rate (line alteration) | 0% | 30.8% |
| p | 0.29 | |

Table 3: Task 3 (needle guidance) - completion time & failure rate

|  | Group A | Group B |
| --- | --- | --- |
| Average completion time (sec) | 73.2 (SD 24.8 sec) | 165.06 (SD 6.4 sec) |
| Median completion time (sec) | 62 | 138.5 |
| Q1 | 61 | 116.25 |
| Q3 | 97 | 158 |
| IQR | 36 | 41.75 |
| p | 0.19 | |

Table 4: Task 4 (simple knot tying) - completion time & failure rate

|  | Group A | Group B |
| --- | --- | --- |
| Average completion time (sec) | 92 (SD 1.4 sec) | 182.42 (SD 19.1 sec) |
| Median completion time (sec) | 82 | 162.5 |
| Q1 | 80 | 118 |
| Q3 | 94 | 216.5 |
| IQR | 14 | 98.5 |
| p | 0.02 | |
| Failure rate (suture placement) | 20% | 3.9% |
| p | 0.31 | |
| Failure rate (margin approximation) | 0% | 11.5% |
| p | 1 | |
| Failure rate (knot slipping) | 0% | 15.4% |
| p | 1 | |

## Figures


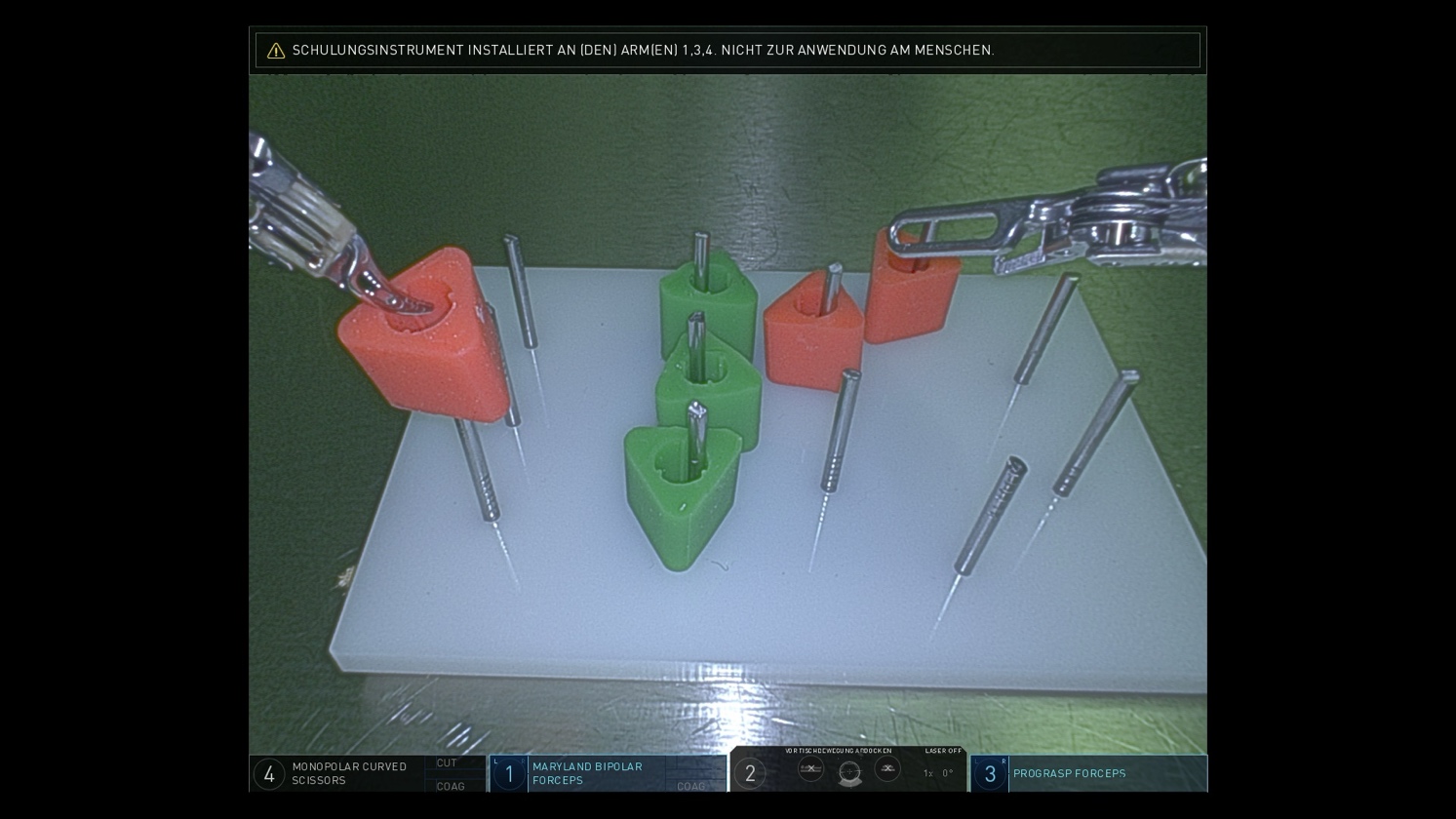


Figure 1: Task 1 (peg transfer)


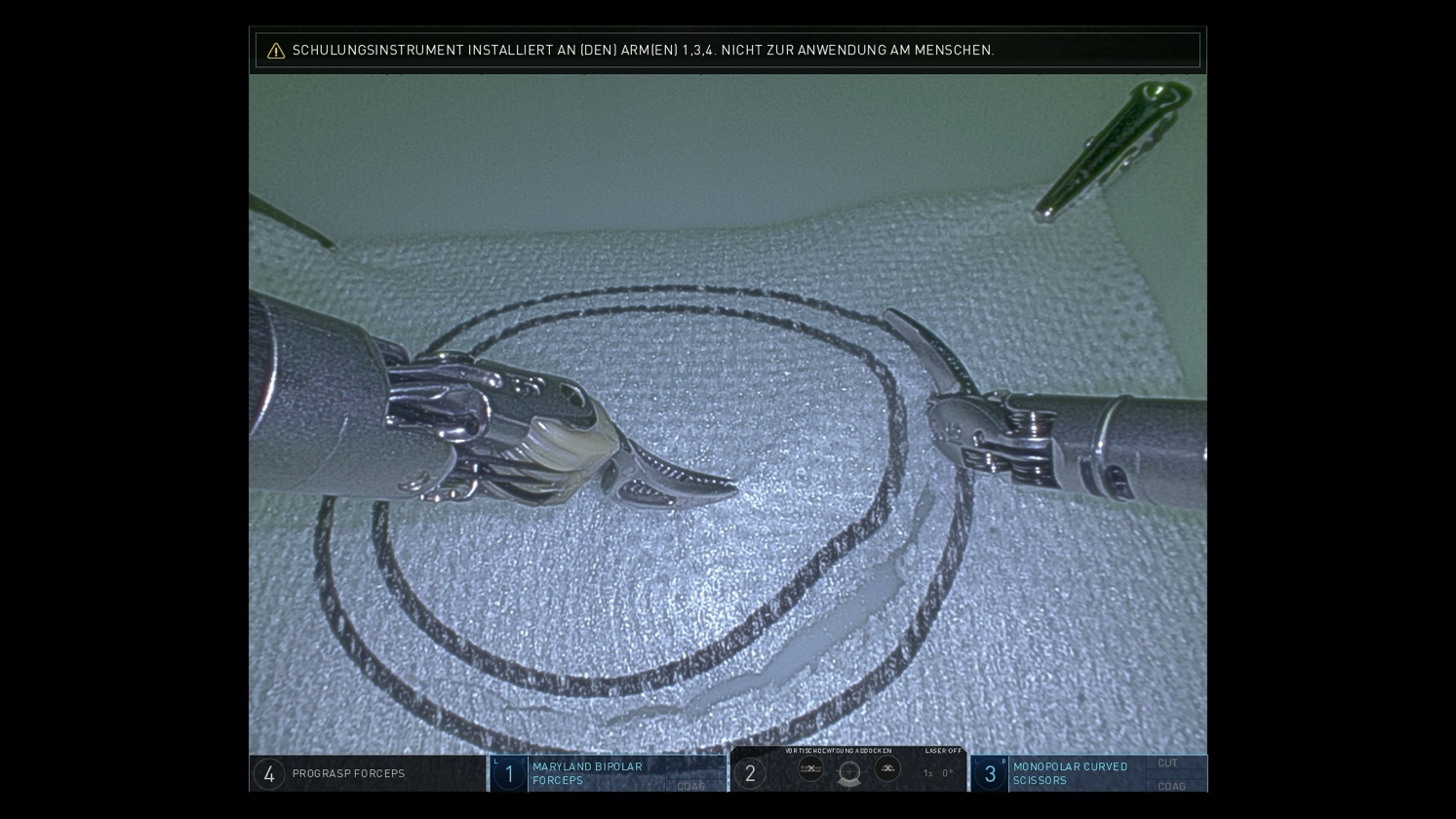


Figure 2: Task 2 (cutting the circle)


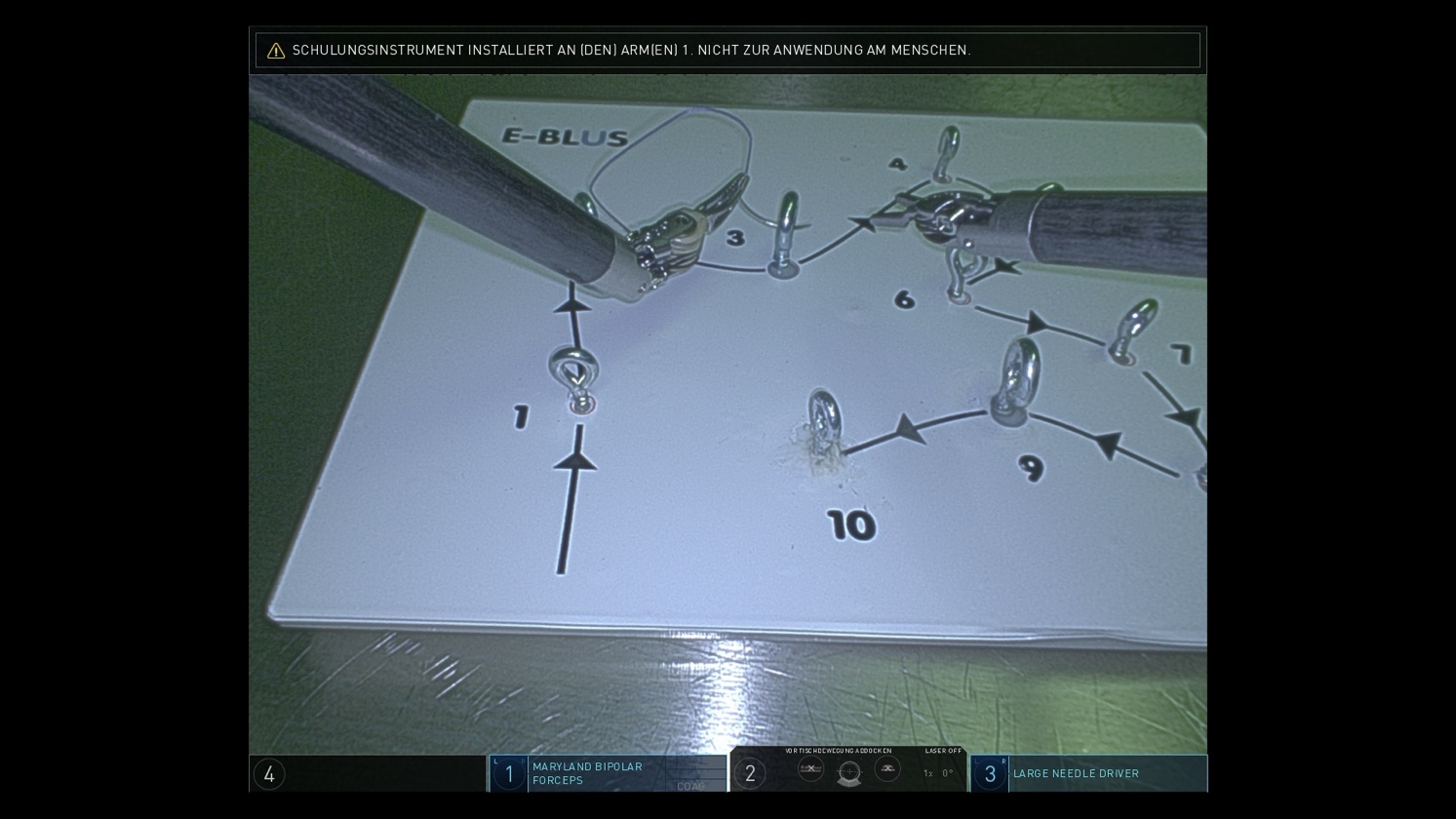


Figure 3: Task 3 (needle guidance)


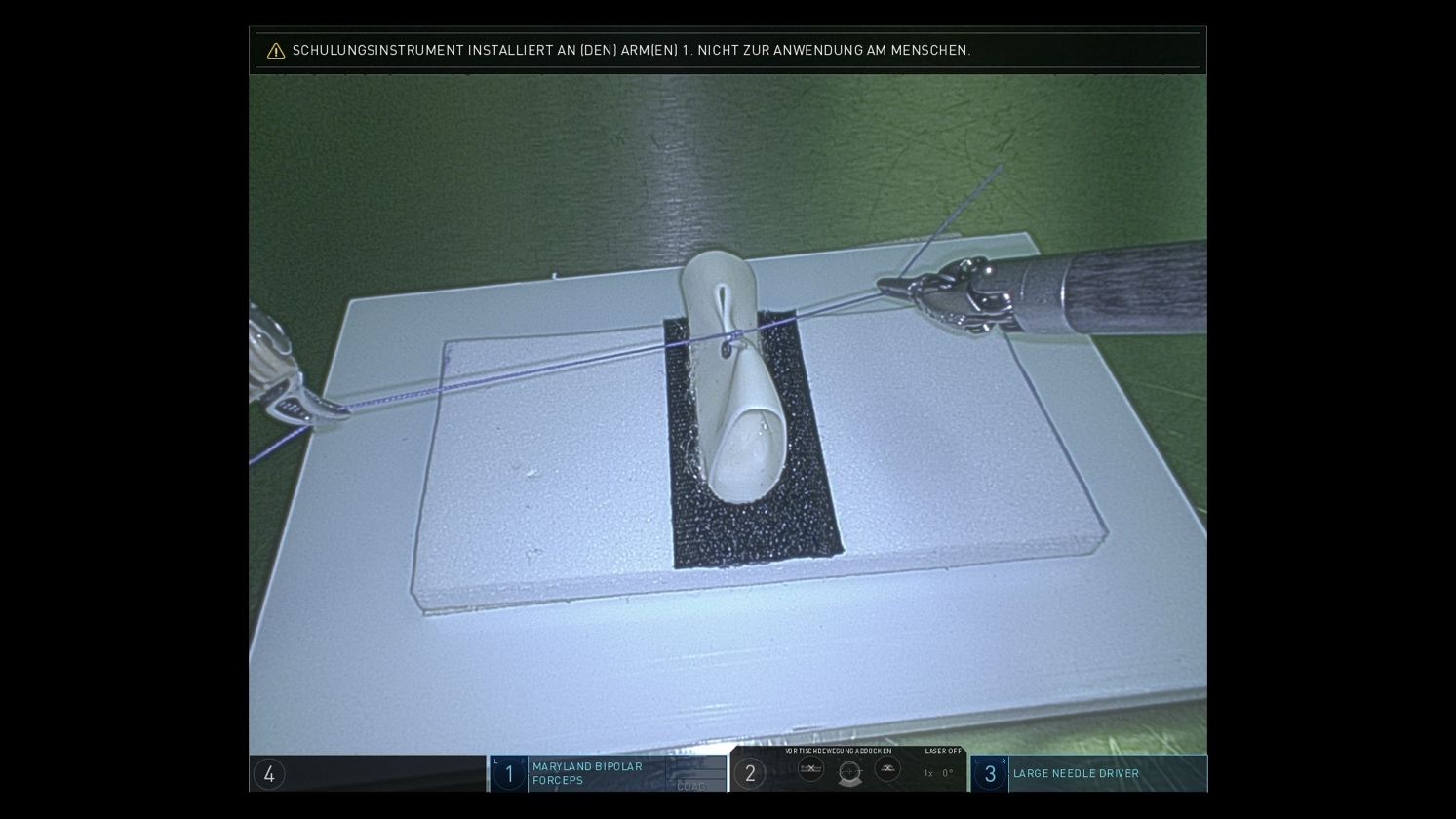


Figure 4: Task 4 (simple knot tying)
